# Incidence and impact of antiplatelet therapy cessation among very older patients with stable coronary artery disease

**DOI:** 10.3389/fphar.2023.1183839

**Published:** 2023-06-05

**Authors:** Xiao Zou, Liang Wang, Sha-Sha Sun, Yi-Xin Hu, Hong-Wei Liu, Hao Wang, Jian Cao, Hong-Bin Liu, Li Fan

**Affiliations:** ^1^ Cardiology Department of the Second Medical Center, National Clinical Research Center for Geriatric Diseases, Chinese PLA General Hospital, Beijing, China; ^2^ The Forth Healthcare Department of the Second Medical Center, Chinese PLA General Hospital, Beijing, China

**Keywords:** stable coronary artery disease, antiplatelet therapy, MACE, bleeding, mortality

## Abstract

**Objectives:** Long-term use of evidence-based antiplatelet therapy is recommended for management of stable coronary artery disease (SCAD). However, non-adherence to antiplatelet drugs is common in older patients. This study aimed to evaluate the incidence and impact of antiplatelet therapy cessation on clinical outcomes of older patients with SCAD.

**Methods:** A total of 351 consecutive eligible very older patients (≥80 years) with SCAD from the PLA General Hospital were included. Baseline demographics, clinical characteristics, and clinical outcomes were collected during follow-up. Patients were divided into cessation group and standard group based on whether discontinuing of antiplatelet drugs. The primary outcome was major adverse cardiovascular events (MACE) and secondary outcomes were minor bleeding and all-cause mortality.

**Results:** A total of 351 participants, with a mean age of 91.76 ± 5.01 years old (range 80–106 years) were included in statistical analysis. The antiplatelet drug cessation rate was 60.1%. There were 211 patients in cessation group and 140 patients in standard group. During a median follow-up of 98.6 months, the primary outcome of MACE occurred in 155 patients (73.5%) in the cessation group and 84 patients (60.0%) in the standard group (HR = 1.476, 95% CI:1.124-1.938, *p* = 0.005). Cessation of antiplatelet drugs increased the rates of angina (HR = 1.724, 95% CI:1.211-2.453, *p* = 0.002) and non-fatal MI (HR = 1.569, 95% CI:1.093-2.251, *p* = 0.014). The secondary outcomes of minor bleeding and all-cause mortality were similar between the two groups.

**Conclusion:** Among very older patients with SCAD, antiplatelet therapy cessation significantly increased the risk of MACE, and continuous antiplatelet drug therapy didn’t increase the risk of minor bleeding.

## Introduction

Coronary artery disease (CAD) is a leading cause of death worldwide. The Chinese Cardiovascular Health and Disease Report 2020 estimated that there were 11.39 million patients diagnosed CAD, which caused 43.81 and 46.66 deaths per 100 individuals in rural and urban areas, respectively (National Center for Cardiovascular Diseases, 2021). Although the mortality and prevalence of CAD have decreased significantly in recent decades, management of stable coronary artery disease (SCAD) remains an important issue.

Oral antiplatelet agents (OAA) including aspirin and a P2Y12 inhibitor (such as clopidogrel) can prevent myocardial infarction (MI), stroke, and death in cardiovascular disease (Antithrom botic Trialists and Collaboration, 2002). Guidelines recommend long-term use of evidence-based medications for management of SCAD (Fihn et al., 2012; Task Force Memberes Montalescot et al., 2013; Section of Interventional Cardiology of Chinese Society of Cardiology et al., 2018). However, adherence is crucial for achieving optimal benefit from antiplatelet therapy and improving clinical outcomes (Bosworth et al., 2011; Reuter et al., 2015). Medication adherence is suboptimal after MI, with a report from 2008 indicating that 1 in 4 patients did not fill their initial cardiac medication prescriptions in the first month after MI, which is associated with higher mortality and cardiovascular events (Jackevicius et al., 2008). However, non-adherence to medications is common among older patients with cardiovascular diseases. Among the reasons for non-adherence in these older patients, independent functional ability non-adherence is common and may represent a significant barrier to optimal adherence.

A previous study showed that patients with a recent interruption in OAA-intake displayed worse clinical outcomes, and OAA withdrawal was found to be an independent predictor of both mortality and bleedings at 30 days (Collet et al., 2004). However, the incidence and impact of recent OAA cessation on clinical outcomes among very older patients with SCAD are still lacking. Therefore, we prospectively investigated whether continuous use or cessation of OAA influences long-term clinical outcomes in very older patients with SCAD.

## Materials and methods

### Study population

A total of 377 patients (age ≥ 80 years) with SCAD were recruited from the Chinese PLA General Hospital from January 2009 to December 2010 and were followed up until December 2020. The inclusion criteria were as follows: 1) age ≥80 years and 2) compliance with SCAD diagnostic criteria. We excluded 26 patients based on the following criteria: 1) major bleeding, cerebrovascular disease, or other unstable conditions over the past one month (*n* = 16) and 2) incomplete data on outcomes during the follow-up period (*n* = 10). Ultimately, a total of 351 patients were found to be eligible for statistical analysis.

### Data collection and definitions

Patient baseline demographics, clinical characteristics, and follow-up outcomes were collected during the follow-up period.

The diagnostic criteria for SCAD were based on the 2013 ESC guidelines on the management of SCAD (Task Force Memberes Montalescot et al., 2013; Section of Interventional Cardiology of Chinese Society of Cardiology et al., 2018) and 2018 Chinese guidelines on the diagnosis and treatment of SCAD (Section of Interventional Cardiology of Chinese Society of Cardiology et al., 2018). Major bleeding corresponded to overt bleeding resulting in death; a bleed in a retroperitoneal, intracranial, or intraocular location; a hemoglobin drop of ≥3 g/dL; or the need for transfusion of ≥2 U blood. Minor bleeding was any clinically important bleeding that did not qualify as major (Collet et al., 2004).

The criteria for antiplatelet therapy cessation were as follows (Levine et al., 2016): aspirin or clopidogrel therapy which had been interrupted for >7 days without a specific reason or without advice from the prescribing practitioner, and no other antithrombotic drugs were adopted during the interruption.

### Follow-up and outcomes

All participants were followed-up until December 2020. Outcome events were recorded through outpatient visits, rehospitalizations, and telephone calls. The primary outcome was major adverse cardiovascular events (MACE), the secondary outcomes were minor bleeding and all-cause mortality. MACE include cardiovascular death, non-fatal MI, restenosis, and angina, as reported in a previous study (Baber et al., 2018).

### Statistical analysis

The baseline characteristics were compared between the standard and cessation groups. Continuous variables were displayed as mean ± SD or median (25th-75th percentile) and compared using the *t*-test or Wilcoxon rank test, as appropriate. Categorical variables are presented as numbers (percentages) and were compared using the chi-square test. Kaplan-Meier survival curves were used to determine the cumulative survival rates at the end of the follow-up period among patients in the two groups.

To explore the association between outcomes and antiplatelet therapy cessation, univariate and multivariate Cox regression analyses were performed to calculate hazard ratios (HRs) with 95% confidence intervals (CIs). The following adjusted covariates were included in the multivariate Cox regression analysis: age, sex, BMI, smoking, drinking, antidiabetic, hypertension, and cancer.

All analyses were conducted using the SPSS Statistics software version 22.0. Statistical significance was set at *p* < 0.05.

## Results

### Baseline characteristics

A total of 351 patients were included in this study; their characteristics are shown in Table 1. Mean age was 91.76 ± 5.01 years (range 80–106 years), and 98.3% of patients were male. A total of 211 (60.1%) patients experienced antiplatelet drug cessation and were placed in the cessation group. Compared with the patients in the cessation group, those in the standard group were more likely to have hypertension and receive antidiabetic therapy. The other baseline characteristics were well balanced between the two groups.

**TABLE 1:** Baseline characteristics of enrolled patients in two groups.

Items	Total NO. (*n* = 351)	Standard group (*n* = 140)	Cessation group (*n* = 211)	*p*-value
Age (years)	91.8 ± 5.0	92.1 ± 5.1	91.5 ± 5.1	0.257
Male, n (%)	345 (98.3)	138 (98.6)	207 (98.1)	0.547
BMI (kg/m2)	23.5 ± 3.3	23.3 ± 3.3	23.6 ± 3.3	0.319
Smoking, n (%)	17 (4.8)	8 (5.7)	9 (4.3)	0.536
Drinking, n (%)	53 (15.1)	22 (15.7)	31 (14.7)	0.793
SBP (mmHg)	133.8 ± 15.6	135.4 ± 15.6	132.8 ± 15.6	0.130
DBP (mmHg)	68.2 ± 9.6	68.6 ± 9.8	67.9 ± 9.4	0.650
Diabetes, n (%)	167 (47.6)	72 (51.4)	95 (45.0)	0.239
Hypertension, n (%)	296 (84.3)	126 (90.0)	170 (80.6)	0.017
Dyslipidemia, n (%)	270 (84.3)	113 (80.7)	157 (74.4%)	0.170
Cancer, n (%)	193 (55.0)	70 (50.0)	123 (58.3)	0.126
Nitrates drugs, n (%)	317 (90.3)	130 (92.9)	187 (88.6)	0.189
lipid-lowering drugs, n (%)	278 (79.2)	117 (83.6)	161 (76.3)	0.100
β-blocker, n (%)	218 (62.1)	94 (67.1)	124 (58.8)	0.113
ACEI/ARB, n (%)	188 (53.6)	82 (58.6)	106 (50.2)	0.125
CCB, n (%)	215 (61.3)	88 (62.9)	127 (60.2)	0.615
Antidiabetics, n (%)	225 (64.1)	80 (57.1)	145 (68.7)	0.027

BMI: body mass index; SBP: systolic blood pressure; DBP: diastolic blood pressure; ACEI/ARB: angiotensin converting enzyme inhibitors/angiotensin receptor blocker; CCB: calcium channel blockers.

### Clinical outcomes

The median follow-up period in our study was 98.6 months (range 2–126 months). The median follow-up period for standard and cessation group were 87.2 months (range 5–125 months) and 88.6 months (range 2–126 months), respectively. There was no significant difference between them during the follow-up period (*p* = 0.401). With regard to the primary outcome of MACE, patients in the cessation group experienced higher MACE rates than those in the standard group (73.5% vs. 60.0%, *p* = 0.008), and also displayed higher rates of non-fatal MI (44.1% vs. 32.9%, *p* = 0.035) and angina (45.0% vs. 34.3%, *p* = 0.045). However, the patients in the two groups displayed similar rates on the secondary outcomes of all-cause mortality and minor bleeding. Table 2 shows all outcomes between the two groups.

**TABLE 2:** Clinical outcomes during the follow-up in the two groups.

Items	Total NO. (*n* = 351)	Standard group (*n* = 140)	Cessation group (*n* = 211)	*p*-value	
MACE	239 (68.1)	84 (60.0)	155 (73.5)	0.008	
Cardiovascular death	50 (14.2)	22 (15.7)	28 (13.3)	0.521	
Non-fatal MI	139 (39.6)	46 (32.9)	93 (44.1)	0.035	
Angina	143 (40.7)	48 (34.3)	95 (45.0)	0.045	
Restenosis	83 (23.6)	35 (25.0)	48 (22.7)	0.627	
Minor bleeding	201 (57.3)	76 (54.3)	125 (59.2)	0.358	
All-cause mortality	256 (72.9)	98 (70.0)	158 (74.9)	0.313	

### Kaplan-Meier Survival Curves

**FIGURE 1:**
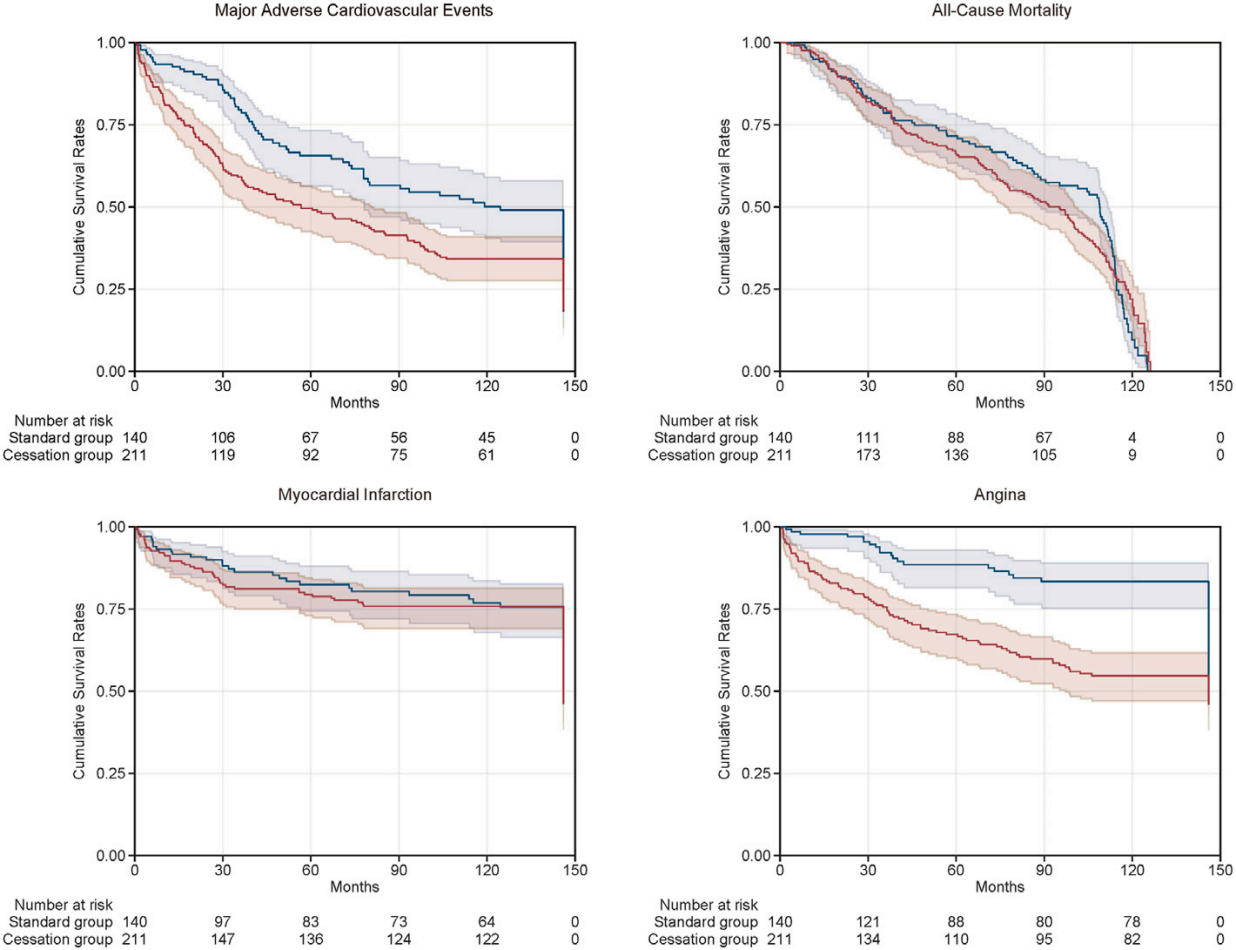
Kaplan-Meier curves of cumulative survival rates of clinical outcomes between standard group (the blue curve) and cessation group (the red curve).

The Kaplan-Meier curves of the two groups are shown in Figure 1. The cumulative survival proportions of MACE at the end of follow-up were statistically different between the standard and cessation groups. Compared with patients in the standard group, those in the cessation group had higher survival rates for non-fatal MI and angina. There were no statistically significant differences in the survival rates of all-cause mortality between the two groups.

### Associations between antiplatelet therapy cessation and outcomes

Multivariate Cox regression analyses were performed to evaluate the association between cessation of antiplatelet therapy and outcomes. We performed the univariate Cox analyses first, and then the multivariate Cox analyses, which were fully adjusted for clinical variables (Table 3); the multivariate Cox analyses showed similar results to univariate models. Patients in the cessation group were more likely to have MACE than those in the standard group (HR = 1.390, 95% CI:1.065-1.813, *p* = 0.015). Similarly, patients in the cessation group were more likely to have non-fatal MI (HR = 1.517, 95% CI:1.065-2.160, *p* = 0.021) and angina (HR = 1.654, 95% CI:1.168-2.341, *p* = 0.005). The interaction between antiplatelet therapy cessation and the secondary outcomes of minor bleeding and all-cause mortality was not statistically significant. These results suggest that cessation of antiplatelet therapy increased the risk of MACE, especially non-fatal MI and angina.

**TABLE 3:** Multivariate cox regression analyses.

Items	Univariate cox regression analysis			Multivariate cox regression analysis		
	HR	95% CI	*P*	HR	95% CI	*P*
MACE	1.390	1.065-1.813	0.015	1.476	1.124-1.938	0.005
Cardiovascular death	0.808	0.462-1.412	0.454	0.825	0.469-1.451	0.505
Non-fatal MI	1.517	1.065-2.160	0.021	1.569	1.093-2.251	0.014
Angina	1.654	1.168-2.341	0.005	1.724	1.211-2.453	0.002
Restenosis	1.043	0.674-1.613	0.850	1.140	0.730-1.781	0.564
Bleeding	1.228	0.923-1.634	0.158	1.277	0.950-1.716	0.105
All cause death	1.020	0.792-1.313	0.881	1.038	0.799-1.349	0.778

Adjusted for baseline variables, including age, gender, body mass index, smoking, drinking, antidiabetic, hypertension, Cancer.

## Discussion

Our study provides insights into the incidence and association between antiplatelet therapy cessation and clinical outcomes in older patients with SCAD. The present study found that 60.1% of patients had experienced antiplatelet therapy cessation. This is the first time that a variable (antiplatelet therapy cessation) has been systematically collected in very older patients with SCAD. Subsequently, we found that antiplatelet therapy cessation appeared to be associated with a higher risk of MACE, especially increased risk of non-fatal MI and angina. However, continuous antiplatelet drug therapy did not increase the risk of minor bleeding in these patients.

Aspirin (or another oral antiplatelet drug) has a protective effect following long-term use in patients at increased risk of occlusive vascular events, including those with ACS or SCAD (Antithrom botic Trialists and Collaboration, 2002). Guidelines have suggested that all patients with SCAD should receive antiplatelet drugs every day for their whole lives unless there are contraindications (Juul-Moller et al., 1992; Committee, 1996; Section of Interventional Cardiology of Chinese Society of Cardiology et al., 2018). Adherence to taking P2Y12 inhibitors is an important determinant of efficacy, with lower rates of compliance being associated with MACE (Lordkipanidze et al., 2020). While aspirin/clopidogrel cessation still occurs for many reasons, with prevalence ranging from 5.4% to 47.4% in previous studies (Collet et al., 2004; Derogar et al., 2013; Baber et al., 2018; Koshy et al., 2022). In our study, all patients were initially administered aspirin or clopidogrel therapy, and 60.1% of patients displayed aspirin/clopidogrel cessation, which was higher than that reported in previous studies. The main reason was that most of the very older patients had comorbidities and dysfunctional activities of daily living.

In the present study, aspirin/clopidogrel cessation patients experienced a higher prevalence of MACE compared to patients who did not cease taking aspirin or clopidogrel (73.5% vs. 60.0%), and also displayed higher rates of non-fatal MI (44.1% vs. 32.9%) and angina (45.0% vs. 34.3%). Ferrari et al. (2005) published 51 cases of coronary events after aspirin cessation. Similarly, they found a higher incidence of ST-segment elevation ACS in aspirin cessation patients. They reported that aspirin cessation in patients with coronary disease may represent a risk for occurrence of new coronary events.

Non-compliance with aspirin/clopidogrel treatment has ominous prognostic implications in subjects at moderate to high risk for CAD. A meta-analysis showed that aspirin non-adherence/withdrawal was associated with a three-fold higher risk of MACE (Biondi-Zoccai et al., 2006). Baber et al. (2018) reported that disruption of antiplatelet therapy was associated with higher risks for MACE among patients with and without chronic kidney disease. A prospective multicenter observational registry study with 5018 PCI-treated patients also found that antiplatelet therapy cessation was associated with a higher risk of MI and MACE (Faggioni et al., 2019). In our study, although the participants were very older patients with SCAD, we found a similar result showing antiplatelet drug cessation was associated with a 1.47-fold higher risk of MACE. The impact of medication on cessation and outcomes was consistent with previous literature.

The prevalence of clinical outcomes in our study was higher than previous studies (Collet et al., 2004; Derogar et al., 2013; Reuter et al., 2015) as the patients in our study differed from those in the other studies. First, the mean age was 91.76 ± 5.01 years, and the median follow-up period of our study was 98.6 months (range 2–126 months); patient age was significantly higher and follow-up time was significantly longer than other studies. Second, most very older patients have more than two comorbidities. These variabilities may have led to the higher rate of clinical outcomes in our study. A similar result was found in our previous study of older patients (Zou et al., 2022).

Baber et al. (2018) published a study on the pattern and impact of various modes of antiplatelet therapy cessation for patients undergoing PCI, and found no significant interaction between the risk of clinical outcomes and each cessation mode. Antiplatelet therapy cessation modes included physician-recommended discontinuation, temporary interruption (≤14 days), and disruption due to bleeding or noncompliance. Hennigan et al. (2022) recently studied the relationship between antiplatelet therapy cessation and clinical outcomes. They found that cessation of any type led to a substantial increase in platelet reactivity, with differential effects on different pathways of platelet activation when aspirin or P2Y12 inhibitors were stopped. Recent years, some studies also evaluate the adherence of other antiplatelet drugs-ticagrelor and prasugrel. Previous study showed 22% of patients with ACS discontinued ticagrelor because of side effects, age >75 years, and bleeding during the follow-up. Koskinas et al. report a study about 1830 patients (17% women, mean age 59 years), they found that prasugrel cessation had occurred in 13.8% of the patients at 1 year, early prasugrel cessation also associated with statistically significant excess in ischemic risk within 1 year after PCI (Koskinas et al., 2018). Further study is required to determine which patients could receive a net benefit from long-term continuation of dual antiplatelet therapy.

## Study limitations

This study has several limitations. Our study was based on patients from a hospital in a single center, and therefore may not be representative of the entire older population. In addition, although the association of antiplatelet therapy cessation with a higher rate of MACE was particularly strong, the special nature of older patients and the presence of unmeasured confounders may have contributed to the strength of the association and survival bias. Lastly, in our retrospective study, we did not classify cessation types due to the insufficient sample size and data limitations. In our future study, we will continue the prospective study to improve the results.

## Conclusion

Among very older patients with SCAD, antiplatelet therapy cessation significantly increased the risk of MACE, and continuous antiplatelet drug therapy did not increase the risk of minor bleeding.

## Data Availability

The original contributions presented in the study are included in the article/supplementary materials, further inquiries can be directed to the corresponding authors.
